# Sexual Dimorphism in the Fibular Extremities of Italians and South Africans of Identified Modern Human Skeletal Collections: A Geometric Morphometric Approach

**DOI:** 10.3390/biology11071079

**Published:** 2022-07-19

**Authors:** Annalisa Pietrobelli, Rita Sorrentino, Stefano Durante, Damiano Marchi, Stefano Benazzi, Maria Giovanna Belcastro

**Affiliations:** 1 Department of Biological, Geological and Environmental Sciences, University of Bologna, 40126 Bologna, Italy; rita.sorrentino2@unibo.it (R.S.); mariagiovanna.belcastro@unibo.it (M.G.B.); 2 Department of Cultural Heritage, University of Bologna, 48121 Ravenna, Italy; stefano.benazzi@unibo.it; 3 IRCCS Azienda Ospedaliero-Universitaria di Bologna Policlinico S. Orsola-Malpighi, 40138 Bologna, Italy; stefano.durante@aosp.bo.it; 4 Department of Biology, University of Pisa, 56126 Pisa, Italy; damiano.marchi@unipi.it; 5 Centre for the Exploration of the Deep Human Journey, University of the Witwatersrand, Johannesburg 2050, South Africa; 6 Natural History Museum of the University of Pisa, 56011 Calci, Italy

**Keywords:** sex determination, human fibula, 3D geometric morphometrics

## Abstract

**Simple Summary:**

The extremities of the fibula may reflect differences between males and females, although so far only few studies included this bone for post-cranial sex assessment. Our work explored shape and size variation between sexes in identified skeletal samples comprising different populations from Italy and South Africa and showed that fibular extremities are significantly smaller, with narrower articular surfaces in females than in males. Consistent sex-related differences are revealed in fibular form and size in Italians but not in South Africans. Potential application in forensic and bioarcheological contexts may benefit from the use of this approach.

**Abstract:**

Fibular metric variations have revealed their potential in distinguishing between males and females; however the fibula remains scarcely analyzed in studies of sexual dimorphism. This work aims at investigating sexually dimorphic features in fibular proximal and distal epiphyses through geometric morphometrics methods. A total of 136 left fibulae, from two Italian and one South African identified skeletal collections were virtually acquired through CT and laser scanning and analyzed using geometric morphometric methods. Statistical analyses were performed on shape, form, and size variables. Results show that fibular epiphyses are smaller with narrower articular surfaces in females than in males in both extremities. Relevant sexual differences emerge in fibular form and size for the two Italian samples but not for the South African one, likely for its small sample size. Discriminant analysis on form principal components (PCs) offers accuracy above 80% when the samples are pooled, and reaches accuracy of 80–93% when the Italian samples are considered separately. However, our method on form PCs was not successful for the South African sample (50–53% accuracy), possibly due to the small sample size. These results show relevant morphological variation in relation to fibular form and size, with a degree of accuracy that indicates the utility of the present method for sexing human fibulae in both forensic and bioarchaeological contexts for Italian samples.

## 1. Introduction

Shape and size of leg bones are widely used in both bioarcheological and forensic contexts as sex indicators, e.g., [1,2,3,4]. Generally, males display larger and more robust tibiae and fibulae, with more robust lower extremities than females [5,6] due to a differentiation in environmental responses, hormonal surges, and physical loading patterns, which contribute to sexual variations of body shape and size/proportions [7].

While the human tibial shape has been extensively studied with biomechanical and morphometric (both traditional and virtual) approaches for sex assessment [3,8,9,10,11], few studies include the fibula. Sex assessment based on multivariate analyses of fibular measurements in populations with different ancestries has reported accuracies of 80–86% [12,13,14,15,16]. Such studies, however, focus solely on gross anatomical fibular features and linear measurements, such as maximum length and maximum diameters, not considering the shape of the extremities. The most often given reason as to why there are few studies focusing on the fibula is the scarcity of fibulae in both forensic and bioarcheological contexts, with only 18–50% of preserved fibular segments of the expected total [17,18,19]. However, it has been reported that for fragments of distal and proximal fibular extremities, the percentage of preservation is between 57–90% and 61–91%, respectively [20].

Singh and Singh [21] have included in their analysis measurements of distal articular surfaces, suggesting that the width of the distal extremity, together with fibular length and mid-shaft circumference, correctly estimated sex with 99% accuracy. In another study, Sacragi and Ikeda [12] focused on the talar articular surface, the malleolar fossa, and the lateral malleolus, and using a population-specific discriminant function, they were able to correctly classify males at 90.1% and females at 91.4%. Naidoo and colleagues [22], in addition, examined different linear and angular dimensions of the talar facet, and found significant variations among males and females, and confirmed consistency among different populations in a South African sample.

Several works examined measurements associated with the fibular nutrient foramen in South African populations [4,22,23]. Fasemore and colleagues [4] found relevant sexual differences among measurements of diameters and circumference taken at the level of the fibular nutrient foramen, with discriminatory cross-validated accuracy of 73% with combined measurements. Interestingly, the authors also highlighted how measurements of distance between the most proximal end of the fibula and nutrient foramen were not sexually dimorphic. The proximal end of the fibula is in fact rarely studied in anthropology for sex determination purposes, and only studies accounting for three-dimensional fibular shape variations included the proximal extremity, along with other tibial and fibular regions, in their analysis [24,25,26,27,28].

Virtual morphometric approaches have the advantage of accounting for the whole bone shape and size variations, overcoming the possible limitations that may arise when focusing on single measurements (e.g., [29]). Prior studies involving three-dimensional statistical shape models (SSM) of fibulae (within the tibiofibular complex), considering both fibular extremities and diaphysis, have found that sex contributes only subtly to the differentiation of fibular morphologies [28]. Similarly, Tümer and colleagues [25] did not find a consistent pattern of shape variation in relation to sex for the fibula. Furthermore, three-dimensional geometric morphometric (3D GM) studies of the whole fibula highlighted little to no morphological differences between the sexes, with the few observed differences mostly occurring in fibular length, but not in fibular shape, which was not sexually dimorphic [30]. Indeed, Maas and Friedling found that fibulae were smaller in females than males. Interestingly, they also describe patterns of sex variations in relation to different ancestries (White, Black, and Coloured individuals, the three main groups present in South Africa). Such differences mostly relate to the contribution of body size to fibular shape among the different ethnicities of their subsamples, since Coloured individuals’ smaller fibulae may possibly reflect the genetic influence of small-bodied Khoisan and Asian groups [30]. While differences in fibular shape emerged among different ancestries, in their study, Maass and Friedling [30] adopted an approach based on fixed landmarks, which captured the whole fibular length, masking more subtle differences in epiphyseal shape and form, and in general is highly influenced by the size component [31,32,33].

In this work we applied for the first time a 3D GM approach that integrates fixed landmarks and sliding (semi)landmarks of curves and surfaces to investigate sexually dimorphic features in the fibular proximal and distal extremities. The study involves three samples from identified modern skeletal collections (19–20th century), two from Italian and one from South African populations. The main goal of this study is to provide a new method for sex assessment of the human fibular extremities based on 3D GM to be applied in forensic and bioarcheological investigations. Based on previous studies on sexual dimorphism in the fibulae, we tested the two following hypotheses:

(a) Fibular epiphyseal form (size + shape) will be sexually dimorphic, likely reflecting functional differences (body shape, size, and proportions), while shape alone will be less informative [30].
(b) Fibular epiphyseal form dimorphism will differ between populations due to size variation (different patterns of epiphyseal size dimorphism due to differentiation of body size among Italian and South African groups), but shape alone will not reveal differences in the sexual dimorphism between the populations [30].

## 2. Materials and Methods

### 2.1. Sample

In this study we analyzed 136 left fibulae (Table 1) belonging to late 19th–early 20th century individuals. The total sample consists of 17 South African individuals of various ancestral origins from the Raymond A. Dart Collection of Human Skeletons at the University of the Witwatersrand (Johannesburg, South Africa) [34], and 119 Italian individuals (Emilia-Romagna, N = 47; Sardinia, N = 72) belonging to individuals from the Modern Human Identified Skeletal Collection of the University of Bologna. Year of death for the whole South African collection spans 1920s to 2000s, while Italian samples include individuals who died between 1898 and 1944 [35]. For each subsample (from now on referred to as population), the sex of the individuals is known from hospital or cemetery records as well as other archival sources (e.g., birth certificates). Age-at-death spans between 17 and 90 years.

The fibulae used in this study were selected for their general good state of preservation and for the absence of pathological markers, with fully fused secondary centers of ossifications [36].

The specimens were either digitized through computed tomography (CT) or laser scanning, as previous studies have shown that digital models of long bones from these two acquisition methods are comparable [37,38,39]. The acquisition of the 3D models of fibulae belonging to the Raymond A. Dart Collection was performed at the Microfocus X-ray Computed Tomography facility of the University of Witwatersrand (Johannesburg, South Africa) on a Nikon Metrology XTH 225/320 LC (Voltage 70 kV, current 120 μA, no filter used, pixel size 120 μm). The acquisition of the fibulae of the Italian collections was carried out at Istituto Ortopedico Rizzoli (Bologna, Italy), utilizing Revolution Discovery CT dual energy, with GSI Revolution and HD Revolution configurations (Voltage 100 kV, current 360 μA, standard filter, slice thickness, and acquisition interval at 0.625 mm, followed by a reconstruction with monochromatic beams at 40 keV, using the “Detail” filter). A subsample of 20 Italian individuals of Bologna was digitized with an ARTEC Space Spider 3D (Luxembourg) laser scanner (3D precision: 0.05 mm; 3D resolution: 0.1 mm) in the Department of Cultural Heritage of the University of Bologna. The generated surface model was exported in STL format (Artec Studio, Luxembourg, 2013).

Data from CT scans were processed in Avizo 9.2 (Thermo Fisher Scientific, Waltham, MA, USA) for image segmentation, utilizing the threshold-based segmentation protocol of half-maximum height (HMH) outlined by Spoor, Zonneveld, and Macho (1993) [40]. We applied the protocol following the modified version detailed by Coleman and Colbert (2007) [41], already used in several applications in anthropology (e.g., [37,42,43]). Then, a surface model was generated (isosurface reconstructions), saved in STL format, and loaded into Viewbox 4 v. 4.0 (dHAL Software, Kifissia, Greece) for landmarking. 

### 2.2. 3D Geometric Morphometric Analysis

**Figure 1:**
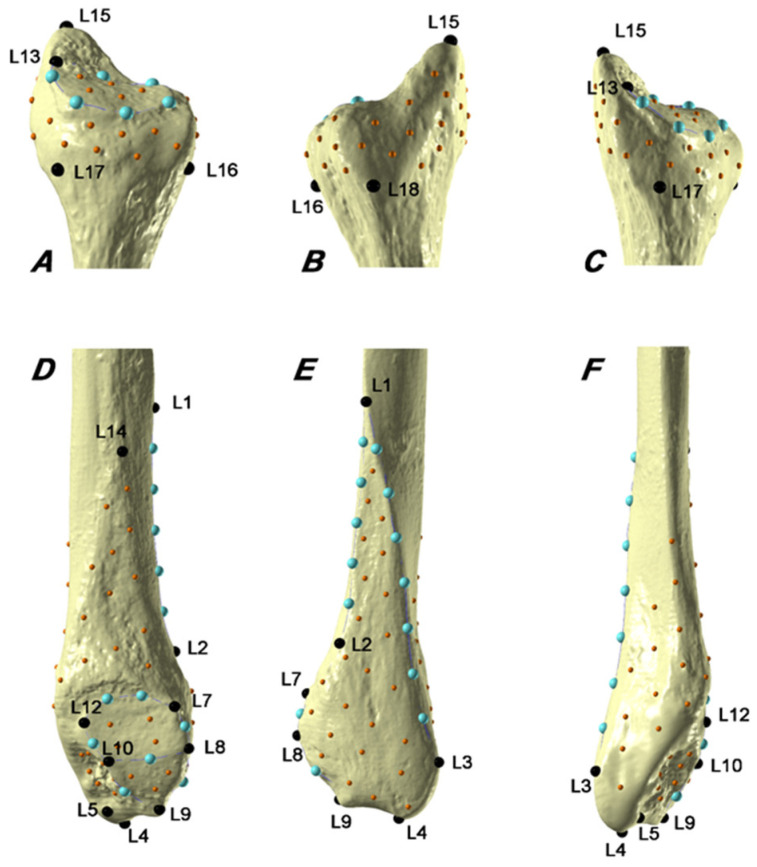
Landmarks (black), curve (light blue), and surface (semi)landmarks (orange) digitized on a left fibula (**A**), proximal fibula, medial view; (**B**), proximal fibula, lateral view; (**C**), proximal fibula, posteromedial view; (**D**), distal fibula, medial view; (**E**), distal fibula, lateral view; (**F**), distal fibula, posterior view. See Table 2 for a detailed description of the anatomical landmarks [37] (license: CC BY 4.0).

A 3D template configuration (Figure 1, Table 2) of 16 fixed landmarks, 25 curve semi-landmarks, and 101 surface (semi)landmarks captured both the distal and proximal extremity of the fibula. The template was created in Viewbox 4, to cover major muscle, ligament, and tendon attachment sites and articular surfaces on the fibula (Figure 1, Table 2). Viewbox 4 software was utilized to apply the configuration to the targets in the sample, with (semi)landmarks sliding on curves and surfaces to minimize thin-plate spline (TPS) bending energy between the targets and the template [44]. Following that, (semi)landmarks can be considered geometrically homologous among specimens [45,46]. The template repeatability (i.e., high intra-observer agreement) and reproducibility with different scanning devices were tested [37]. This work revealed that for this template (1) the intra-observer error is negligible, (2) and that the 3D-GM comparisons of specimens scanned with different scanning devices are not influenced by repeating the template on the same individual (intra-observer error). Landmarks and (semi)landmarks raw coordinates used to describe the specimens of the study are available in Tables S1 and S2. After importation of raw coordinates in R (version 4.0.3) [47], the set of (semi)landmarks of proximal and distal epiphysis was separated into two different datasets (i.e., a dataset of Cartesian coordinates for the distal and a dataset for the proximal fibular extremity). Each dataset was subjected to a further sliding step against recursive updates of the Procrustes consensus, while two different Procrustes superimpositions were computed, allowing the conversion of raw coordinates into standardized, scaled, centered, and oriented shape coordinates (i.e., Procrustes coordinates) via Generalized Procrustes Analysis (GPA) [31,44] using the R package “geomorph” version 3.3.2 [48]. Centroid size (CS) for each extremity, which is the square root of the summed squared distances between each (semi)landmark and the centroid of the (semi)landmark configuration, was also calculated and used as a proxy of the size of the distal and proximal extremities [44].

Procrustes coordinates were then subjected to two separate principal component analyses (PCA) to explore shape variations based on different sex groups for both distal and proximal extremities separately, both considering each population separately and the pooled sample [49]. Two form-space (i.e., shape plus size) PCAs were computed by augmenting the Procrustes shape coordinates of each dataset of Procrustes coordinates by the natural logarithm of CS (lnCS) [50]. Visualization of extreme shape and form changes along the principal axes and means based on sex and population groups were obtained by TPS deformation [51] of the Procrustes grand mean shape surface utilizing the R package “Morpho” v. 2.8 [49]. Linear correlation among CS and the scores along the first two shape principal components of proximal and distal epiphyses were assessed by a Pearson’s correlation test, to assess whether the distribution of PC scores in the shape-space PCA plot is influenced by size. The assumptions of this test (linearity, continuous and paired variables) were all met by our variables.

Procrustes ANOVA was adopted to test shape differences among sexes, both considering each population and the pooled sample, utilizing Procrustes distances among specimens and using a residual randomization procedure (RRPP = T, iterations = 1000), with the R package “geomorph” version 3.0.7 [48]. Differences in size among sexes and populations were evaluated considering CS through ANOVA and subsequent post hoc tests and were visualized in relative box plots. This permutation design allows one to avoid Type I error and normality violations. 

Allometric trajectories were estimated for both fibular epiphyseal extremities following Sorrentino and co-workers (2020) [29], by computing a multivariate regression of shape and form variables (using all the PCs) on CS. Then, all the obtained coefficients (slope and intercept) were utilized to compute a permutation test (N = 1000) on lengths (i.e., magnitude of the variability) and angles to assess significant differences (i.e., *p* < 0.05) among trajectories by populations and sexes group trajectories [52]. The assumptions of this multivariate regression (independence; linearity; normality; homoscedasticity) were all met by our variables (PCs).

Finally, linear discriminant analysis (LDA) with leave-one-out cross-validation testing assessed classification accuracy of sex based on shape variations using shape and form PC scores and centroid size to classify the specimens (i.e., either male or female). Selection within the first 10 PCs to include into LDA was based on optimization between the amount of explained variance, considering 70% as threshold [53] and the highest accuracy values obtained. Again, the assumptions of LDA (independence; linearity; normality; homoscedasticity) were all met by our variables (PCs).

## 3. Results

### 3.1. Pooled Sample

**Figure 2:**
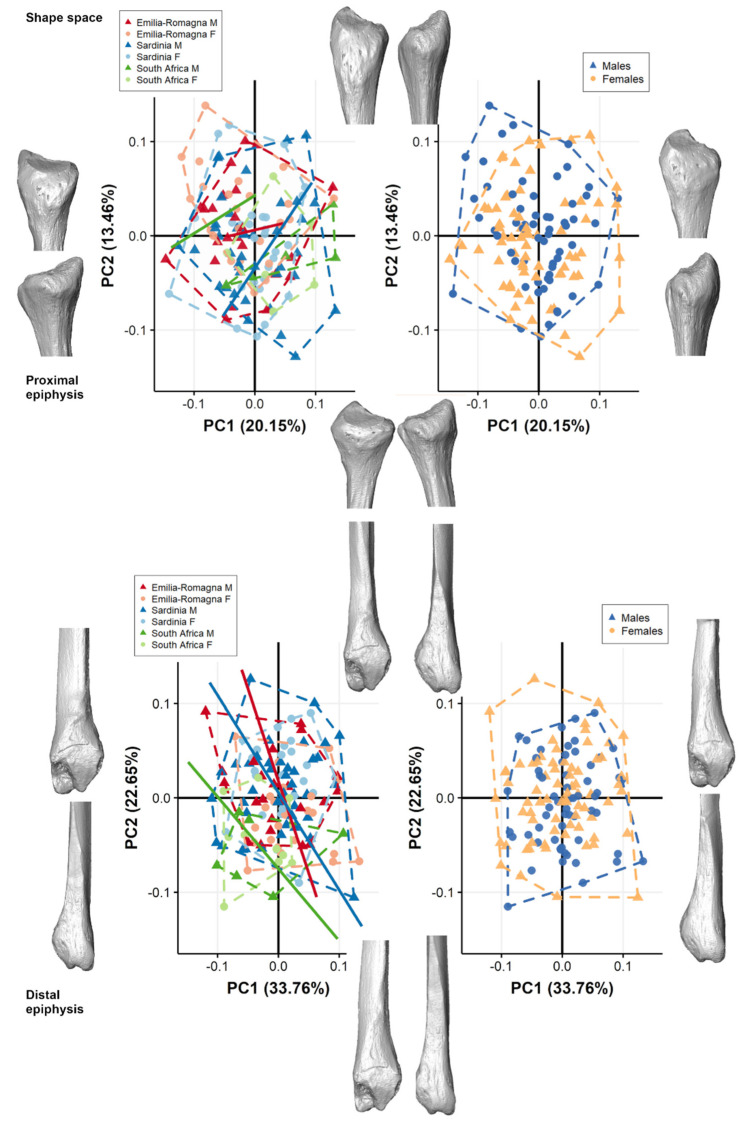
Scatterplot of shape-space PC scores for both proximal and distal epiphyses computed considering all populations pooled together, grouped by population (on the **left**) and by sex (on the **right**). For both proximal (**above**) and distal (**below**) extremities, positive and negative extreme shape variations along PC1 (**right** and **left**) and PC2 (**top** and **bottom**) are represented. Lines represent allometric trajectories for all populations. Allometric trajectories for ER (red line), SAR (blue line), and SA (green line) are represented.

Italian populations (i.e., Emilia-Romagna, ER; and Sardinia, SAR) mostly overlap in shape space with each other and with South African individuals (SA) for both proximal and distal epiphyses. Additionally, no clear separation is present among individuals according to sex groups for any populations (Figure 2). For the proximal epiphysis, the first two principal components (PCs) accounts for 33.61% of total variance (PC1: 20.15%; PC2: 13.46%), while for the distal epiphysis the variance explained by the first two PCs is 56.41% (PC1: 33.76%; PC2: 22.65%). For both extremities, the first two PCs do not show a distinction among males and females (*p* > 0.05), with comparable males and females with extreme shapes (Figure 2). Few to no morphological changes are seen in the extreme shape along PC1 and PC2 axes for both extremities (Figure S1) and are mostly related, for the proximal epiphysis, to a more pronounced styloid process towards positive scores and a more laterally protruding insertion of the m. fibularis longus towards negative scores of PC1 and PC2; for the distal epiphysis, to a more elongated and less pronounced area of tibiofibular syndesmosis towards positive PC1 scores and negative PC2 scores, and to a more craniocaudally elongated subcutaneous triangular surface along negative scores on PC1 and PC2. For the proximal epiphysis, no significant correlation is present between the first two PCs scores and CS. In contrast, for the distal epiphysis, a Pearson’s correlation test shows that PC2 is significantly negatively correlated with CS (r = −0.565; *p* < 0.001), indicating that static allometry could account for morphological differences in shape along this axis. Angles of allometric trajectories between population groups do not differ significantly (Table 3, Figure 2). 

**Figure 3:**
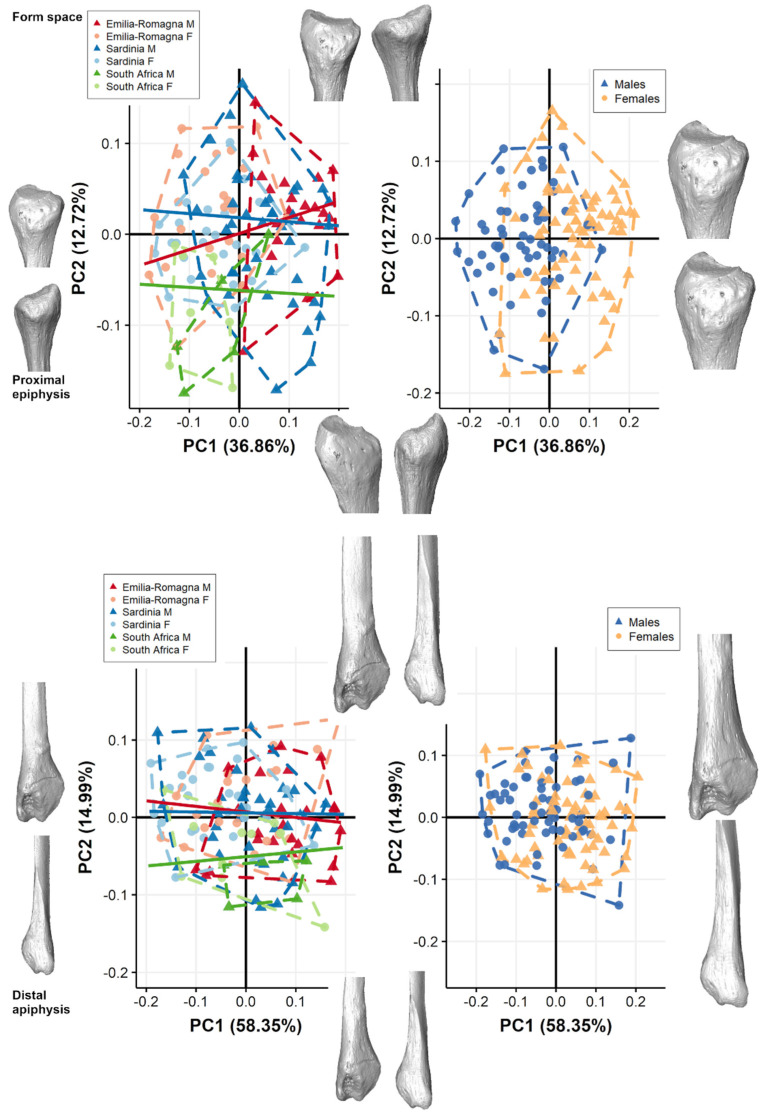
Scatterplot of form-space PC scores for both proximal and distal epiphyses computed considering all populations pooled together, grouped by population (on the **left**) and by sex (on the **right**). For both proximal and distal extremities, positive and negative extreme form variations along PC1 (**right** and **left**) and PC2 (**top** and **bottom**) are represented. Lines represent allometric trajectories for all populations. Allometric trajectories for ER (red), SAR (blue), and SA (green) are represented.

The form-space PCA plots of both extremities (Figure 3) show a clear separation among the sexes, with males plotting towards positive scores along PC1 when considering all populations pooled and grouped by sex. The first two PCs account for 49.05% of total variance for proximal epiphysis (PC1: 36.86%; PC2: 12.72%), while for distal epiphysis the first two components account for 73.34% of total variance (PC1: 58.35%; PC2: 14.99%). For both extremities, PC1 significantly contributes to the separation among males and females (proximal epiphysis: ANOVA; PC1: F-value = 88.39, *p* < 0.001; distal epiphysis: ANOVA; PC1: F-value = 18.26, *p* < 0.001), accounting mainly for size variation (i.e., CS). No distinction is present between sexes along PC2 or PC3 (*p* > 0.05). Generally, fibular epiphyses are smaller in females than in males, with narrower articular surfaces (Figure S2). For proximal epiphyses, males (PC1 positive) have a more pronounced styloid process, and a bulkier, laterally protruding fibular head compared to females. For distal epiphyses, males (PC1 positive) show a longer insertion of tibiofibular interosseous ligament, site of the tibiofibular-syndesmosis, longer subcutaneous triangular surfaces, peroneal grooves, protruding malleoli, attachments for calcaneofibular ligament and anterior and posterior talofibular ligaments, and a deeper and wider malleolar fossa, attachment for the transverse tibiofibular and posterior talofibular ligaments in comparison to females. Angles of allometric trajectories between groups do not differ significantly according to different populations (Table 3). 

**Figure 4:**
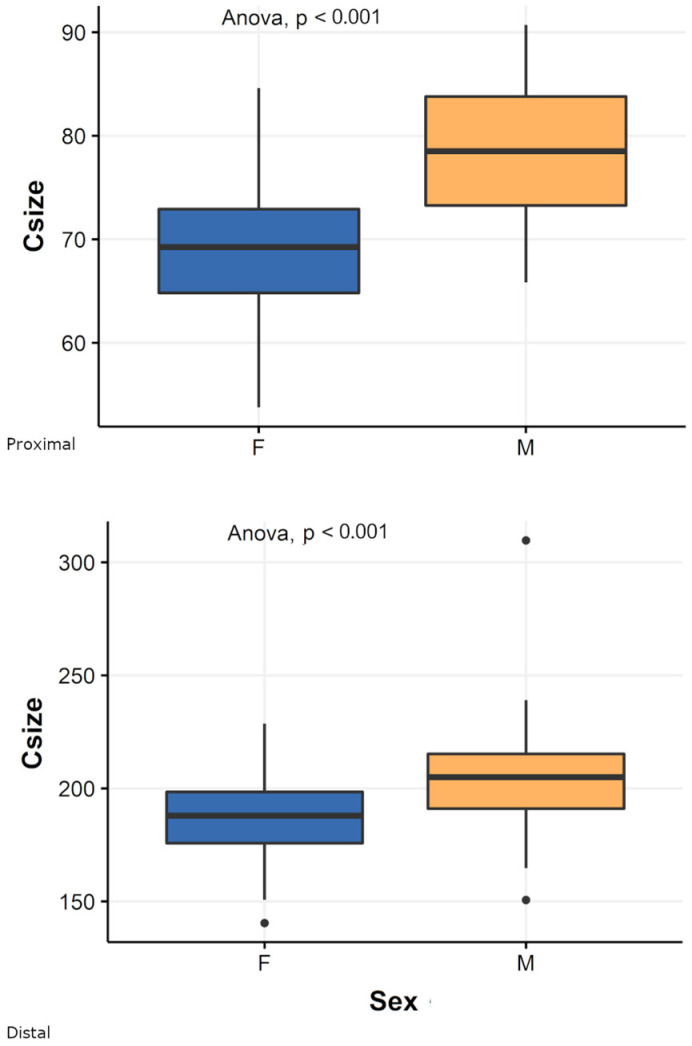
Box plot of centroid size (Csize) of proximal (**above**) and distal (**below**) fibular epiphyses for the pooled sample by sex. M = males; F = females. Black lines are the medians, boxes represent the interquartile ranges, whiskers the non-outliers range and filled circles the outliers.

For both extremities, CS is significantly different between pooled males and females (proximal epiphysis: ANOVA; F-value = 89.39, *p* < 0.001; distal epiphysis: ANOVA; F-value = 7.38, *p* < 0.001) (Figure 4).

Cross-validation LDA of the pooled sample reaches accuracy above 80% when using the first six form space PCs (76.87% of variance explained) and the first four form space PCs (83.38% of variance explained) for proximal and distal epiphyses, respectively (Table 4). Using CS, accuracy is between 68 and 76%, considering both epiphyseal ends. Fewer individuals are correctly classified according to sex when using shape-space PCs, with accuracy of 57–62% using 10 PCs (82.05% and 85.12% of variance explained for proximal and distal epiphysis, respectively) (Table 4).

### 3.2. Separate Populations

**Figure 5:**
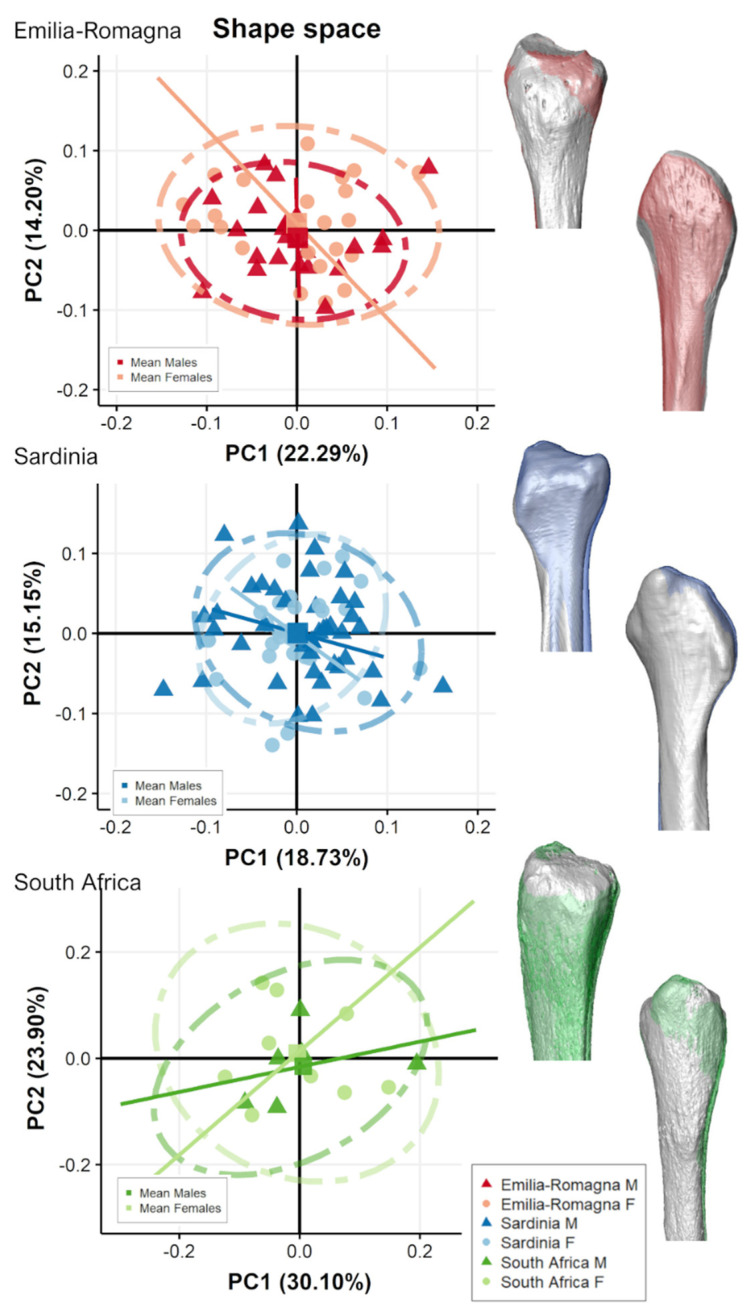
Scatterplot of shape-space PC scores of proximal fibular epiphyses for the two Italian (Emilia-Romagna and Sardinia) and the South African populations, divided by sex and presenting mean male (colored) and female (grey) shape variation along the PC axes, represented in the scatterplot as squared dots, in medial (**top**) and lateral (**bottom**) views. Lines represent allometric trajectories for males and females for each population.

**Figure 6:**
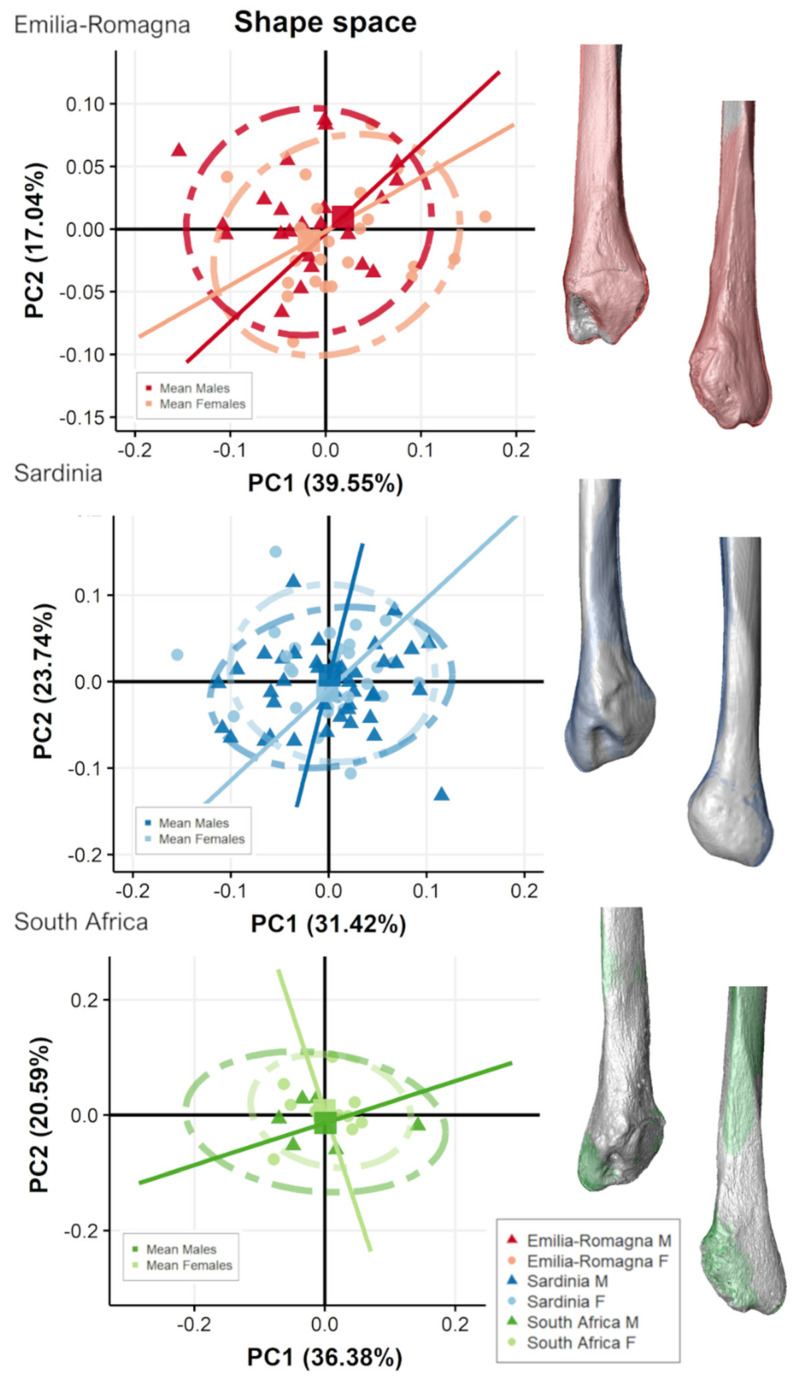
Scatterplot of shape-space PC scores of distal fibular epiphyses for the two Italian (Emilia- Romagna and Sardinia) and the South African populations, divided by sex groups and presenting mean male (colored) and female (grey) shape variation along the PC axes, represented in the scatterplot as squared dots, in medial (**top**) and lateral (**bottom**) views. Lines represent allometric trajectories for males and females for each population.

Figure 5 and Figure 6 show the shape-space PCA plots of proximal and distal fibular extremities, respectively, for each population grouped by sex and their related mean shape. In all populations and for both fibular extremities, PCs scores are not significantly correlated (−0.5 < r < 0.5) with CS, except for the negative correlation of PC2 in SA for distal epiphysis (r = −0.706, *p* = 0.002)

In general, only subtle shape morphological changes are seen among mean shapes of males and females of each population and are mostly related to a slightly wider anteroposterior expansion, with longer malleoli in males in comparison with females, which possess, for ER, a shallower malleolar fossa for distal epiphyses. In shape-space, indeed, PC scores along the first three PC axes do not contribute to the separation among males and females in any of the three populations (ANOVA, *p* > 0.05) for both proximal and distal epiphyses, except for the distal extremity of ER which shows weak significant differences between males and females (ANOVA, PC1: F-value = 4.21, *p* = 0.046). Angles of shape allometric trajectories do not differ significantly according to sex, considering each population separately (Table 5). 

**Figure 7:**
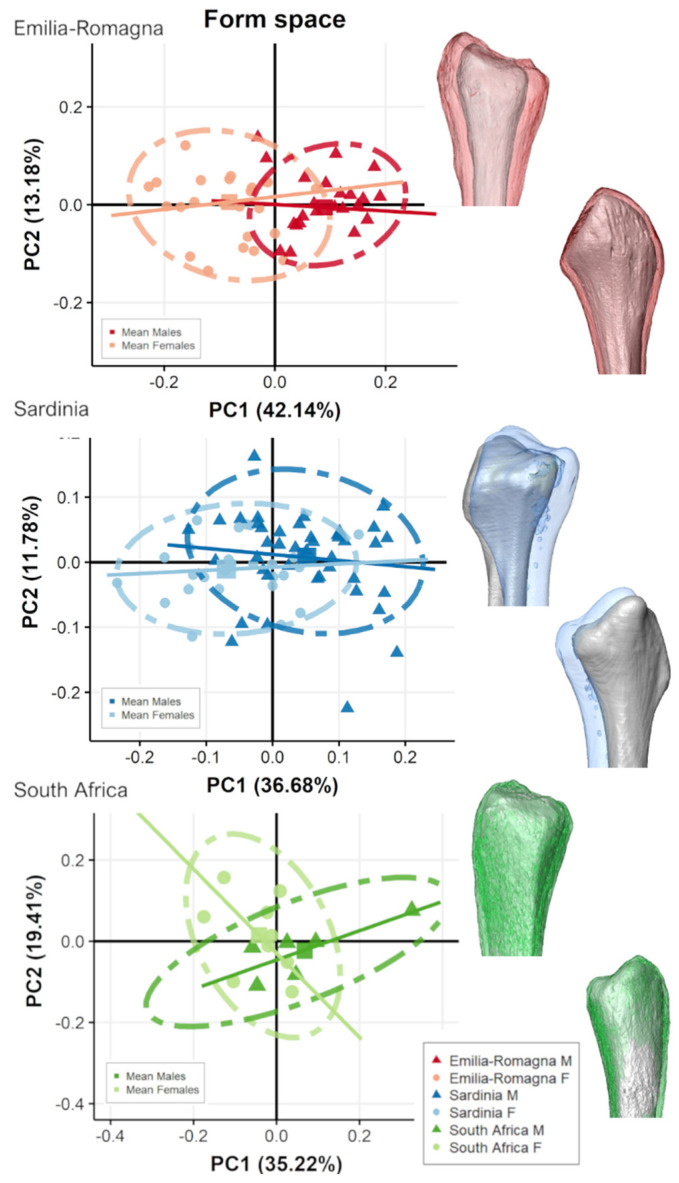
Scatterplot of form-space PC scores of proximal fibular epiphyses for the two Italian and the South African populations, divided by sex groups and presenting mean male (colored) and female (grey) form variation along the PC axes, represented in the scatterplot as squared dots, in medial (**top**) and lateral (**bottom**) views. Lines represent allometric trajectories for males and females for each population.

**Figure 8:**
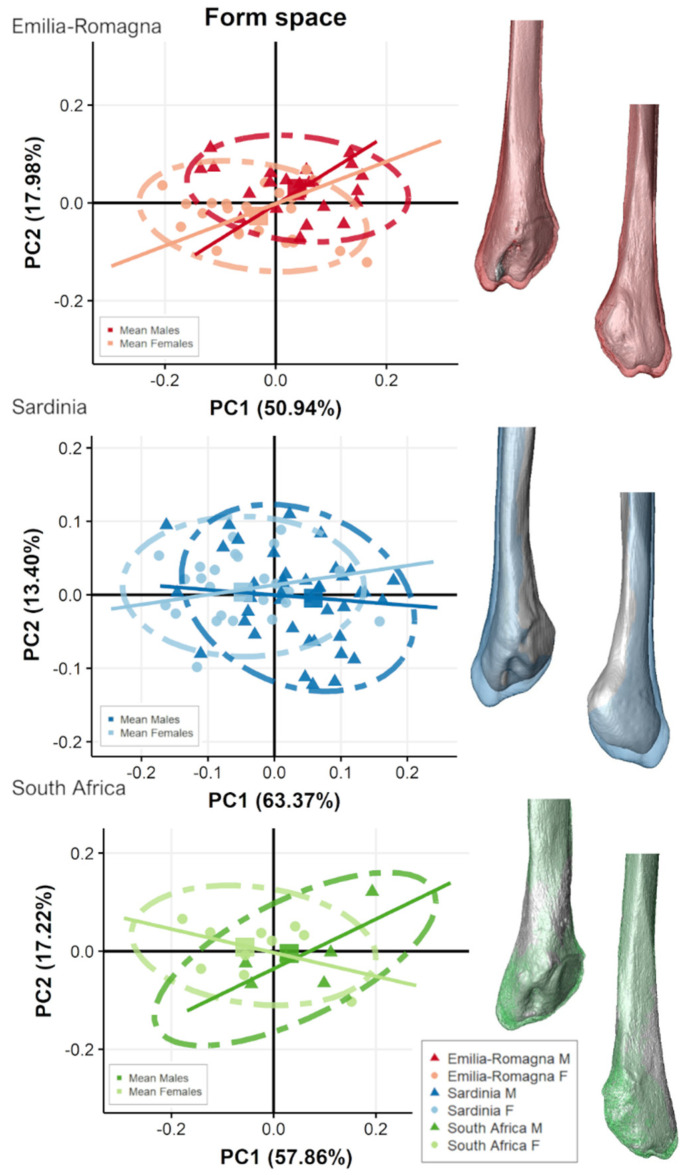
Scatterplot of form-space PC scores of distal fibular epiphyses for the two Italian and the South African populations, divided by sex groups and presenting mean male (colored) and female (grey) form variation along the PC axes, represented in the scatterplot as squared dots, in medial (**top**) and lateral (**bottom**) views. Lines represent allometric trajectories for males and females for each population.

Figure 7 and Figure 8 show the scatterplot of form-space PCA plot of proximal and distal fibular extremities, respectively, for each population grouped by sex and their related mean shape. For all populations, PC1 shows the main separation between sexes as accounting for size variation (i.e., CS represented by PC1). 

For the two Italian populations, males plot towards PC1 positive scores and females plot towards negative scores (Figure 7 and Figure 8), although slightly overlapping, especially on distal fibula extremity (Figure 8). ANOVAs show significant separation (*p* < 0.05) along PC1 between sexes for both proximal and distal epiphyses for the two Italian populations but not for South Africans. For distal epiphyses, moreover, Italian ER population also presents a significant separation according to sex along PC2 (ER: PC2, F-value = 15.54, *p* < 0.001). For proximal epiphysis (Figure 7), Italian males show wider fibular heads with more antero-posteriorly expanded tibiofibular articular surfaces and a more pronounced styloid process than females. In addition, males possess an area of insertion of knee collateral ligaments, m. biceps femoris, and fibularis longus, which is more postero-laterally protruding than in females, especially evident in SAR population. For distal epiphysis (Figure 8), Italian males exhibit more mediolaterally expanded proportions, with longer and pronounced malleoli, attachment for calcaneofibular ligament and anterior and posterior talofibular ligaments, and a deeper and wider malleolar fossa, attachment for the transverse tibiofibular and posterior talofibular ligaments, in comparison to females. In contrast, South African males do not show such marked pattern of form variation, with comparable mean form morphologies seen in both sexes. Angles of form allometric trajectories do not differ significantly according to sex considering each population separately (Table 5). 

**Figure 9:**
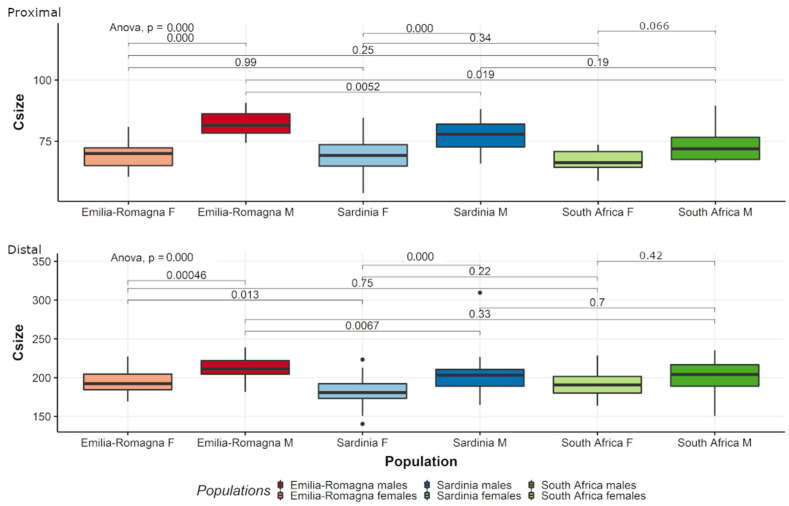
Box plot of centroid size (Csize) of proximal (**above**) and distal (**below**) fibular epiphyses all populations by sex. M = males; F = females. Black lines are the medians, boxes represent the interquartile ranges, whiskers the non-outliers range, and filled circles the outliers.

For both extremities, CS is significantly different between males and females of the two Italian populations (ANOVA, ER, and SAR: *p* < 0.05), but does not significantly discriminate between sex groups in South Africans (Figure 9). For proximal epiphyses, females of all populations do not differ among one another, and neither do SA and SAR males, who differ from ER but not between each other. For distal epiphysis, ER females and males differ from their SAR counterparts, while both Italian males and females do not differ significantly with their SA equivalents.

Table 6 shows cross-validation LDA accuracy percentages of sex determination by populations for both fibular epiphyses. Shape PCs provide less discriminatory power in all three populations, with accuracy percentages of correct sex estimation using proximal epiphysis spanning 40–54% in Italians and 60% in South Africans. Similarly, for distal epiphyses, accuracy is lower for shape PCs, ranging 51–72%. As regards form PCs and considering both extremities, in ER the first 3–5 PCs (76–77% of variance explained) provide 89–93% accuracy in sex determination; in SAR the first 4–6 PCs (71–84% of variance explained) provide 80–83% accuracy in sex determination. Accuracy percentages of LDA on form PCs on South Africans are lower (including both epiphyses, 50–53%, with 77–79% of variance explained considering the first four PCs). Centroid size separates sexes—considering both epiphyses—with 69–87% accuracy for Italian populations, and with 66–68% accuracy for South African populations.

## 4. Discussion

The aim of this study was to highlight patterns of sexually dimorphic form variations in the fibular proximal and distal epiphyses in individuals of three different populations from Italy and South Africa belonging to identified modern skeletal collections (19th–20th century), utilizing a 3D GM approach [54]. Our study provides a new method for sex estimation on the fibula, potentially applicable in bioarcheology [20], where this method may find primary implementation, and forensics, offering moderate to high accuracy (80–93% for Italian populations) for identification in case of the recovery of isolated fragments of this bone.

We expected the presence of variations of fibular epiphyseal size in relation to sex. Results from our analyses support a distinction among sexes, mainly more robust epiphyses in males than in females and especially in the Italian samples. Our second hypothesis was that little sex variation would have been present in the shape of the fibular epiphyses in relation to sex, depending on different ancestries and mostly referring to different patterns of epiphyseal size in different populations, due to a differentiation of body size among Italian and South African groups. Our results support the former point and provide only partial support to the latter one. In fact, the form and size of fibular extremities significantly distinguish between sexes in the Emilia-Romagna (ER) and Sardinia (SAR) populations but not in the South African population. Overall, our results show that fibular extremities account for subtle shape changes, while significant form and size differences are present between sexes, even with differences related to ancestry. The sex determination method provided in this paper, based on cross-validation LDA on form PCs, provides accuracy above 80% when the samples are pooled and reaches accuracy of 80–93% when Italian populations are considered separately. However, the method was not successful for the South African sample (50–53% accuracy). 

Our results are consistent with previous investigation on sex determination using the fibula, adopting both traditional metrics [4,12,16,22,55] and virtual methods [25,26,27,28,30]. Specifically, we obtained similar results as previous studies that utilized 3D GM methods on the whole fibula [30], which highlighted different degrees of size dimorphism between males and females, but no significant shape differences between the sexes were observed once size information is removed. 

In our study, morphological differences of both extremities in form space suggest that females possess smaller articular surfaces, shorter and narrower malleoli, and shallower malleolar fossas, with subsequently reduced areas of insertion of knee and ankle collateral ligaments, and less robust muscle insertions for the m. fibularis longus, m. biceps femoris, and interosseous ligament distal attachment (Figure 3, Figure 7, Figure 8, and Figure S2). This indeed may reflect a sex-specific pattern of knee [56,57,58,59] and ankle/foot morphology and posture [29,60,61], and in general supports the existence of sex-segregated patterns of lower limb muscle activation in the different sexes [62,63,64,65]. Regarding the ankle, previous studies have demonstrated sex-related differences in anatomical and biomechanical features of the joint [59]. Adjei, Nalam, & Lee [66], congruently with Trevino and Lee [67], found that ankle stiffness varied significantly between sexes, both along the sagittal and frontal planes (therefore concurring in dorsiflexion/plantarflexion and inversion/eversion, respectively), with greater joint stiffness in females while both quiet standing and muscle activation occurred. The authors explain their findings with the greater passive resistance of females to a greater range of motion, lower elastic modulus, and higher ligamentous laxity [68,69,70] than males, which by contrast possess more leg muscle mass [71] and a higher muscle and cortical bone cross-sectional area in the whole lower limb [7,9,72] than females. Females have an increased range of motion in the sagittal plane of rearfoot and midfoot, peak plantarflexion angle of rearfoot, peak dorsiflexion and abduction angle of midfoot, as compared with males [73]. Females also possess greater ligamentous laxity, resulting subsequently in this increased mobility of the ankle joint [68,74]. Indeed, our results show that females have shallower malleolar fossae with reduced area of insertion of ankle stabilizers (transverse tibiofibular and posterior talofibular ligaments), less protruding malleoli with reduced area of insertion of ankle collateral ligaments (anterior, posterior talofibular, and calcaneofibular ligaments), and less robust muscle insertions for the m. fibularis longus, the main evertor of the ankle (Figure 3, Figure 7, Figure 8 and Figure S2). This result seems also to indicate sex-specific differences in structural properties of ankle stabilizers, as differences in tendon tissue properties of the leg have already emerged [70]. 

Regarding the knee, important differences among the sexes have been established in morphology and kinematics [75]. El Ashker and colleagues [57] found that males have higher functional hamstring-to-quadriceps strength ratios than females, suggesting possible quadriceps dominance in females or greater contribution of the hamstrings in males than in females, either in motion or during foot contact with the ground, stabilizing the joint angle. Their findings seem congruent with our result, showing a protruding area of attachment of m. biceps femoris in the proximal fibular extremity in males (Figure 3, Figure 7, Figure 8 and Figure S2). While the specific relationship between a muscle and its tendon can change depending on several factors (e.g., muscle/tendon specificity, loading degree, age), in our sample, such variations are minimal, since all individuals possess similar activity levels (i.e., sedentary lifestyle [76], and age variations in the samples are similar (Table 1). Therefore, tendon size could be used to assess muscle size [54]. Finally, we did not find significant shape/form sex-related variation regarding articular orientation in agreement with previous studies on sex variations of the bones of the leg [28], but see also [77]. 

Our results show only weak sex-related differences in fibular muscle and ligament insertions when size is excluded from analysis (Figure 2, Figure 5, Figure 6 and Figure S1). It is interesting to notice that studies have found higher mean torque of all major muscle groups in the lower extremity in men (women < 62–70% of men), but when measurements are standardized by size (i.e., bone mass index or body weight), no significant differences in strength, endurance, or torque between sexes have been found [78]. However, when body surfaces and dimensions are evaluated aside from muscle attachments, the relationship between sex, shape, and size is more complex: Wunderlich and Cavanagh [79] stressed that female lower limbs are not solely scaled-down versions of the male lower limb. Particularly, the lateral side of the foot, directly involved in eversion, showed marked sex-related differences even after standardization of size [60]. Indeed, our analysis indicates consistent differences between males and females in size, as sexes differ according to CS in the two Italian populations and when the sample is pooled. According to our results, fibulae of males possess larger linear dimensions than females, due to a generally larger body size (regardless of body mass), possibly due to a relatively longer pubertal growth period, and the subsequent development of larger bones and muscles under the influence of sex genes, sex steroids (androgens and estrogens), and other hormones, such as growth hormone (GF) and insulin-like growth factor 1 (IGF1) which, concurring with mechanical loading, may further contribute to the development of skeletal sexual dimorphism [80,81,82]. Indeed, bone growth is differentiated between sex groups, mostly evident during puberty but to a lesser extent also apparent in early childhood [83,84,85], and this concurs with the observed greater cortical bone plasticity in males, modelled by greater muscle mass during ontogeny, determining greater long bone lengths and breadths, also already observed for the fibula in a similar sample [86]. During puberty, males develop higher peak bone mass, greater bone size, and, ultimately, a stronger skeleton than females [87,88], with different skeletal maturation timing for both fibular extremities according to sex [55]. Our results are also congruent with the already observed different sizes in males and females for the whole size of the tibiofibular [27,28] and with prior assessments on the fibula alone [30]. 

The present study highlights the importance of a population-based approach for sexing the human fibula, as our results point to a different degree of sexual dimorphism in populations with different ancestries (Figure 7, Figure 8 and Figure 9). Maass and Friedling [30] found that Coloured males and females differed significantly from Black and White males and females, with the latter groups not differing from one another, and they interpreted their results in light of the genetic contribution of small-bodied groups within the Coloured group. However, in their case, males and females had significantly different size within the three populations, considered separately. Our results comparing different ancestries, on the other hand, show that sexual variations in fibular extremities are not strictly related to size: in fact, while Sardinians and South Africans have similar (smaller) fibular epiphyseal sizes, the former show a difference in size among sexes, while the latter do not (Figure 9). Some fluctuations in the degree of sexual dimorphism among different populations are expected and could be ascribed to either proximity among individuals of the same population (i.e., response to nutritional stress or overall improvements in the environment) or ultimate causations (i.e., selection and genetic adaptation to a variety of ecological, social, or economic factors) [89,90]. 

Fibular extremities appear more dimorphic in the Italian samples than in the African one, likely as the result of the small sample size characterizing the South African sample or for other reasons which are not explored in this study. This result needs to be further investigated by increasing the sample size and including populations with different ancestry, subsistence strategies, and lifestyles that may lead to differences in the shape of the bones [76]. However, the 80–93% accuracy in sex determination observed for the Italian samples shows the potentiality of the method presented in this study and is congruent with similar discriminant functions provided by studies on fibular sexual dimorphism [4,12,13,14,15,16,22,23,91], mostly reaching the threshold of 80% suggested for sex estimation using long bones [92]. Indeed, when form PCs do not differ significantly among the sexes, accuracy percentages are much lower. When size is considered aside from shape variations (CS), accuracy percentages are quite reduced (69–87%), suggesting that the interaction between size and shape (i.e., form) mainly accounts for sex differences in the human fibula. 

The main limitation of this study is the low sample size of the South African sample, which may have influenced the lack of significance in all comparisons performed in our study. The small sample size is due to limited resource availability at the time of the sample collection. 

Caution should therefore be taken in drawing conclusions on the lack of sexual dimorphism within this sample, as a larger number of individuals may indeed reveal sex-related patterns that are here undetected. A further confounding factor in the South African sample may be the mixed origin of the populations included in this study, whose different body may translate into masking the effect of size sexual dimorphism [34]. Moreover, other factors may have contributed to the lack of sex variation in fibula, such as genetic background, environmental changes, and changes in socioeconomic and health conditions, as already observed in the larger bones of the lower limb [93]. Even though previous GM results on the fibula indicate a lack of sexual dimorphism in the fibula [30], the different methodology here employed (with sliding semi-landmarks instead of only fixed landmarks) could better detect subtle differences. Further studies are therefore necessary to evaluate in more detail this aspect in the South African population. Another limitation of the methodology here proposed is its time-consuming programming phase, which could limit its application in forensic contexts despite its accuracy. 

## 5. Conclusions

In this study, we provided a novel methodology that can be used to determine sex in bioarcheological and, to a lesser extent, forensic investigations using the proximal and distal extremities of the human fibula, rarely analyzed in anthropology. To our knowledge, this is the first study that addresses fibular epiphyseal shape, form, and size in relation to sexual dimorphism, adopting a 3D GM method that includes both fixed landmarks and sliding (semi)landmarks, which together account for detailed morphological variations of fibular extremities. Our work included three different populations, two from Italy and one from South Africa, belonging to identified modern skeletal collections (19th–20th century). 

In comparison to other sex determination methods based on fibular linear measurements, our method, despite being more time-consuming, provides an objective, more reliable, and repeatable tool for investigating this district in Italian populations when fibular extremities are found to be isolated and intact, which could be useful, especially for bioarcheological purposes but also in the forensic field. This method could also be integrated into a wider, accurate evaluation of the whole tibiofibular complex for sex determination, alongside the tibia.

The results of the 3D GM study of the proximal and distal extremities of the fibula showed that the fibula can be used to assess sex with accuracy ranging from 80% to 93% in the two Italian populations. The differences mainly consist in larger and more robust epiphyses in males than in females, with females showing narrower articular surfaces, shorter and narrower malleoli, shallower malleolar fossa and peroneal groove, and less protruding muscle insertions for the m. fibularis longus, m. biceps femoris, and interosseous ligament distal attachment. Such distinct form morphology may reflect a sex-specific pattern of knee and ankle/foot posture, with distinctive lower limb morpho- functional aspects in relation to sex. Our results also highlight the importance of a population-based approach to sex determination of the human fibula, as evidenced by our finding that sexual dimorphism can be determined for the two Italian populations but not for the South African one, reasonably related to its small sample size. This may suggest a different degree of sexual dimorphism in populations with different ancestries, which is not strictly related to size variations. However, when the sample is pooled, our sex estimation method based on cross-validation LDA on form PCs gives accuracy above 80%, which is lower when considering the size alone and the shape PCs, suggesting that the interaction of size and shape is the pattern that mainly reflects sexual dimorphism in the human fibula.

## Figures and Tables

**Table 1:** Sample composition by populations, age range, and sex.

Population	Age Range	Females	Males	Total
Emilia-Romagna (MHISC UBOL *)	18–84	24 (18%)	23 (17%)	47
Sardinia (MHISC UBOL)	17–88	29 (21%)	43 (31%)	72
South Africa (Dart Collection)	20–90	10 (7%)	7 (5%)	17
**Total**	17–90	63 (46%)	73 (54%)	136

* Modern Human Identified Skeletal Collection of the University of Bologna.

**Table 2:** Fibular landmarks and (semi)landmarks identification, definition, and number [37] (license: CC BY 4.0).

Landmarks	Definition	
**L1**	Point where the *fibular anterolateral border* divides into two ridges: the proximal apex of the *subcutaneous triangular surface* (STS)	
**L2**	Most medial point of the medial border of the STS	
**L3**	Most lateral point of the lateral border of the STS	
**L4**	Most distal point of the *lateral malleolus* in anterior view	
**L5**	Most distal point of the posterior border of the *malleolar fossa*	
**L7**	Most anterior point on the anterior border of the *proximal fibular-talar articular facet* (PAF)	
**L8**	Point between the anterior border of PAF and the *anterior border of distal fibular-talar articular facet* (DAF)	
**L9**	Most distal point of DAF	
**L10**	Most proximal point on the posterior border of DAF	
**L12**	Most posterior point of the proximal border of PAF	
**L13**	Most proximal point of *proximal tibiofibular articular facet*	
**L14**	Most proximal point of *interosseous tibiofibular ligament* (ILA) insertion	
**L15**	Most proximal point on styloid process of fibular head in medial view	
**L16**	Most anteroproximal point on anterior border in medial view (above fibular neck)	
**L17**	Most posteroproximal point on posteromedial border in medial view (above fibular neck)	
**L18**	Most posteroproximal point on posterior border in lateral view (above fibular neck)	
**Curves**	**Definition**	**N of semi-landmarks**
**C_1->2**	Medial border of the *subcutaneous triangular surface* (STS)	5
**C_1->3**	Posterior border of the STS	7
**C_7->8**	Anterior border of the *proximal fibular-talar articular facet* (PAF)	1
**C_8->9**	Anterior border of the *distal fibular-talar articular facet* (DAF)	1
**C_9->10**	Posterior border of the DAF	1
**C_10->12**	Posterior border of the PAF	1
**C_8->10**	Border between the PAF and DAF	1
**C_13->13**	Outline of *proximal tibiofibular articular facet*	6
**C_7->12**	Proximal border of the PAF	2
**Surfaces**	**Definition**	**N of semi-landmarks**
**SSML_malleolar fossa**	Surface of the *malleolar fossa*, attachment site of the *transverse tibiofibular* and *posterior talofibular ligaments*.	7
**SSML_ILA**	Attachment surface of *interosseous tibiofibular ligament* and part of *interosseous membrane* (ILA)	13
**SSML_fibular groove**	Groove for tendons of m. *peroneus longus* and m. *tertius* and attachment site of *posterior tibiofibular ligament*	13
**SSML_STS**	*Subcutaneous triangular surface* (STS)	24
**SSML_FiTal1Ar**	Proximal *fibular-talar articular facet* (PAF)	4
**SSML_FiTal2Ar**	Distal *fibular-talar articular facet* (DAF)	3
**SSML_head**	Proximal *tibiofibular articular surface*	5
**SSML_prox_ep**	Surface of *proximal epiphysis*	32

**Table 3:** Allometric trajectory comparisons among different populations computed with the pooled sample for proximal and distal fibular epiphyses in shape and form space. *α* (°) indicates the angle of divergence of each trajectory in pairwise comparisons, with relative *p*-value (*p*).

Pooled Sample			
	Allometric Trajectories		
	***Shape***		
		***α* (°)**	***p***
*Proximal*	**ER-SAR**	95.95	0.190
	**ER-SA**	54.24	0.987
	**SAR-SA**	84.93	0.548
*Distal*	**ER-SAR**	29.75	0.381
	**ER-SA**	35.99	0.792
	**SAR-SA**	35.84	0.520
	***Form***		
		***α* (°)**	***p***
*Proximal*	**ER-SAR**	16.92	0.234
	**ER-SA**	24.38	0.305
	**SAR-SA**	25.54	0.440
*Distal*	**ER-SAR**	9.47	0.426
	**ER-SA**	11.96	0.799
	**SAR-SA**	12.05	0.580

**Table 4:** Sex determination accuracy percentages obtained by cross-validation LDA on the pooled sample (Emilia-Romagna, Sardinia, and South Africa) for shape and form PC scores (PCs) and centroid size (CS) of proximal and distal epiphyses.

	Pooled Sample		
	***Shape***		
	**PCs**	**N**	**%acc**
*Proximal*	10	76/133	57
*Distal*	10	85/163	62
	***Form***		
	**PCs**	**N**	**%acc**
*Proximal*	6	107/133	80
*Distal*	4	111/136	81
	***Csize***		
		**N**	**%acc**
*Proximal*		102/133	76
*Distal*		93/136	68

**Table 5:** Allometric trajectory comparisons of males and females computed for each population separately, for proximal and distal fibular epiphyses in shape and form space. *α* (°) indicates the angle of divergence of each trajectory in pairwise comparison, with relative *p*-value (*p*).

*Separate Populations*				
	Allometric Trajectories M-F			
	***Shape***			
	**Proximal**		**Distal**	
	***α* (°)**	***p***	***α* (°)**	***p***
**ER M-F**	76.44	0.543	31.39	0.548
**SAR M-F**	54.38	0.978	36.31	0.347
**SA M-F**	122.44	0.05	89.34	0.242
	***Form***			
	***α* (°)**	***p***	***α* (°)**	***p***
**ER M-F**	28.33	0.604	14.43	0.658
**SAR M-F**	16.17	0.99	13.92	0.381
**SA M-F**	140.21	0.195	43.16	0.215

**Table 6:** Sex determination accuracy percentages obtained by cross-validation LDA on each population for shape and form PC scores (PCs) and centroid size (CS) of proximal and distal epiphyses.

	Emilia-Romagna			Sardinia			South Africa		
	***Shape***			***Shape***			***Shape***		
	**PCs**	**N**	**%acc**	**PCs**	**N**	**%acc**	**PCs**	**N**	**%acc**
*Proximal*	10	19/47	40	7	39/71	54	4	9/15	60
*Distal*	5	24/47	51	7	52/72	72	5	9/16	56
	***Form***			***Form***			***Form***		
	**PCs**	**N**	**%acc**	**PCs**	**N**	**%acc**	**PCs**	**N**	**%acc**
*Proximal*	5	42/47	89	6	49/71	80	4	8/15	53
*Distal*	3	44/47	93	4	60/72	83	4	8/16	50
	***CS***								
		**N**	**%acc**		**N**	**%acc**		**N**	**%acc**
*Proximal*		41/47	87		49/71	69		10/15	66
*Distal*		34/47	72		50/72	69		11/16	68

## Data Availability

Raw landmarks coordinates and sample list of individuals included in the present study are available as Supplementary Materials. All R scripts utilized for the analyses are available upon request to the corresponding authors.

## Supplementary Materials

The following supporting information can be downloaded at: https://www.mdpi.com/article/10.3390/biology11071079/s1. Table S1: Landmarks and (semi)landmarks raw coordinates. Table S2: Sample list. Figure S1. Shape-space morphings along PC1 and PC2 for both proximal (on the left) and distal epiphyses (on the right) considering extreme positive and negative shapes. Figure S2. Form-space morphings along PC1 and PC2 for both proximal (on the left) and distal epiphyses (on the right) considering extreme positive and negative forms.
