# Supplementary material for: Sexual Dimorphism in the Fibular Extremities of Italians and South Africans of Identified Modern Human Skeletal Collections: A Geometric Morphometric Approach
Source: Biology (Basel). 2022 Jul 19;11(7):1079. doi: 10.3390/biology11071079 (PMC9312998; doi:10.3390/biology11071079)
Supplement: Supplementary file 1 [file biology-11-01079-s001.zip › Supplemetary Information.pdf]

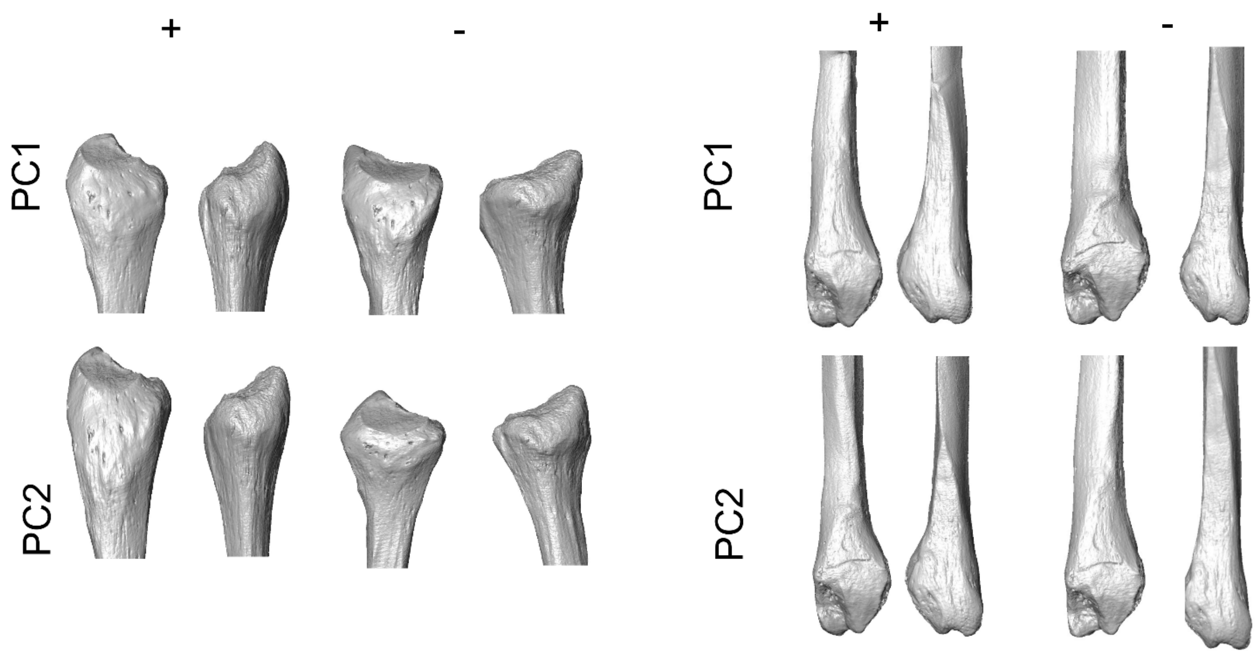

Figure S1. Shape-space morphings along PC1 and PC2 for both proximal (on the left) and distal epiphyses (on the right) computed considering all populations pooled together considering extreme positive and negatives shapes.

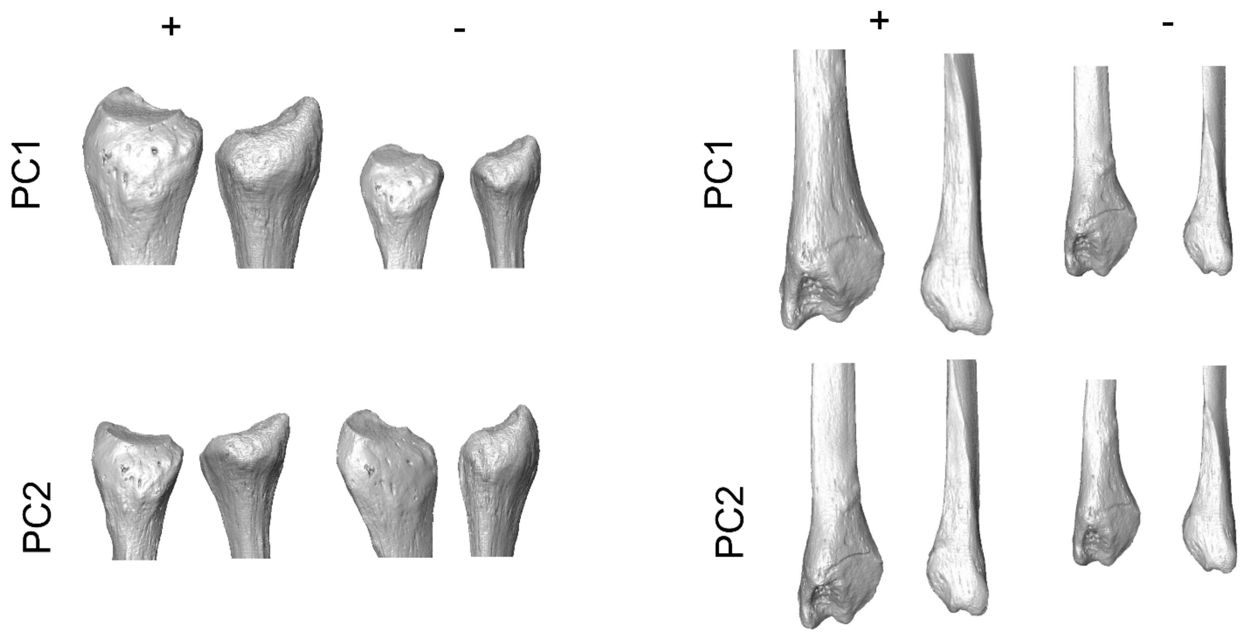

Figure S2. Form-space morphings along PC1 and PC2 for both proximal (on the left) and distal epiphyses (on the right) computed considering all populations pooled together considering extreme positive and negatives forms.
